# Evidence that myoglobin Po_2_ is a noninvasive measure of in vivo skeletal muscle mitochondrial Po_2_ during exercise in humans

**DOI:** 10.1152/function.022.2026

**Published:** 2026-10-01

**Authors:** Ryan M. Broxterman, Bradley A. Ruple, Peter D. Wagner, Russell S. Richardson

**Affiliations:** ^1^Department of Internal Medicine, University of Utah, Salt Lake City, Utah, United States; ^2^Geriatric Research, Education, and Clinical Center, VA Medical Center, Salt Lake City, Utah, United States; ^3^Department of Medicine, University of California, San Diego, California, United States; ^4^Department of Nutrition and Integrative Physiology, University of Utah, Salt Lake City, Utah, United States

**Keywords:** ^1^H-MRS, maximal oxygen uptake, mitochondria, skeletal muscle

## Abstract

Muscle mitochondrial partial pressure of oxygen (${\mathrm{Pmito}}_{{\text{O}}_{2}}$) is a key determinant of skeletal muscle oxygen consumption (${\dot{\text{V}}\text{O}}_{2}$), metabolism, gene regulation, and adaptation in health and disease. Yet ${\mathrm{Pmito}}_{{\text{O}}_{2}}$ remains difficult to measure directly in vivo in humans during exercise. Myoglobin-associated Po_2_ ($P\text{M}{\text{b}}_{{\text{O}}_{2}}$), measured noninvasively using proton magnetic resonance spectroscopy, may provide a measure of ${\mathrm{Pmito}}_{{\text{O}}_{2}}$ during maximal exercise in humans because Mb is coupled to mitochondrial oxygen availability, and $P\text{M}{\text{b}}_{{\text{O}}_{2}}$ falls in the low Po_2_ range expected for ${\mathrm{Pmito}}_{{\text{O}}_{2}}$ in vivo. However, it remains unknown whether $P\text{M}{\text{b}}_{{\text{O}}_{2}}$ during maximal exercise varies systematically with muscle maximal oxygen consumption (${\dot{\text{V}}\text{O}}_{2\text{max}}$), as would be expected if $P\text{M}{\text{b}}_{{\text{O}}_{2}}$ reflects ${\mathrm{Pmito}}_{{\text{O}}_{2}}$ in vivo. We analyzed data from five initially sedentary males before and after 8 weeks of single-leg knee-extensor exercise (KE) training. During maximal KE under 0.12, 0.21, and 1.00 fractions of inspired oxygen (${F\text{l}}_{{\text{O}}_{2}}$), we measured $P\text{M}{\text{b}}_{{\text{O}}_{2}}$ using myoglobin desaturation measured by proton magnetic resonance spectroscopy and muscle ${\dot{\text{V}}\text{O}}_{2\text{max}}$ from leg blood flow and the arterial–femoral venous O_2_ content difference. Across ${F\text{l}}_{{\text{O}}_{2}}$ conditions, muscle ${\dot{\text{V}}\text{O}}_{2\text{max}}$ varied systematically with $P\text{M}{\text{b}}_{{\text{O}}_{2}}$ both before and after training, consistent with O_2_-dependent mitochondrial respiration. When interpreted within a canonical hyperbolic framework, the ${\dot{\text{V}}\text{O}}_{2}$-$P\text{M}{\text{b}}_{{\text{O}}_{2}}$ relationship yielded low apparent mitochondrial P_50_ values that were broadly consistent with values reported for human skeletal muscle-isolated mitochondria studied in vitro. A complementary linear P_50_ analysis that did not assume a hyperbolic relationship supported the same interpretation. Together, these findings provide data-supported, proof-of-concept evidence that $P\text{M}{\text{b}}_{{\text{O}}_{2}}$ reflects in vivo ${\mathrm{Pmito}}_{{\text{O}}_{2}}$ during maximal exercise in humans.

**NEW & NOTEWORTHY** Direct measurement of mitochondrial partial pressure of oxygen (${\mathrm{Pmito}}_{{\text{O}}_{2}}$) in exercising human skeletal muscle remains difficult. By integrating noninvasive myoglobin Po_2_ ($P\text{M}{\text{b}}_{{\text{O}}_{2}}$) and invasive maximal muscle oxygen consumption (${\dot{\text{V}}\text{O}}_{2\text{max}}$) measurements across graded oxygen availability, we found that $P\text{M}{\text{b}}_{{\text{O}}_{2}}$ behaves as expected for ${\mathrm{Pmito}}_{{\text{O}}_{2}}$ during maximal exercise in humans. These findings provide proof-of-concept evidence that $P\text{M}{\text{b}}_{{\text{O}}_{2}}$ reflects ${\mathrm{Pmito}}_{{\text{O}}_{2}}$ in vivo, potentially enabling new insights into metabolism, hypoxia-sensitive signaling, and exercise capacity in health and disease.

## Graphical abstract

**Figure:**
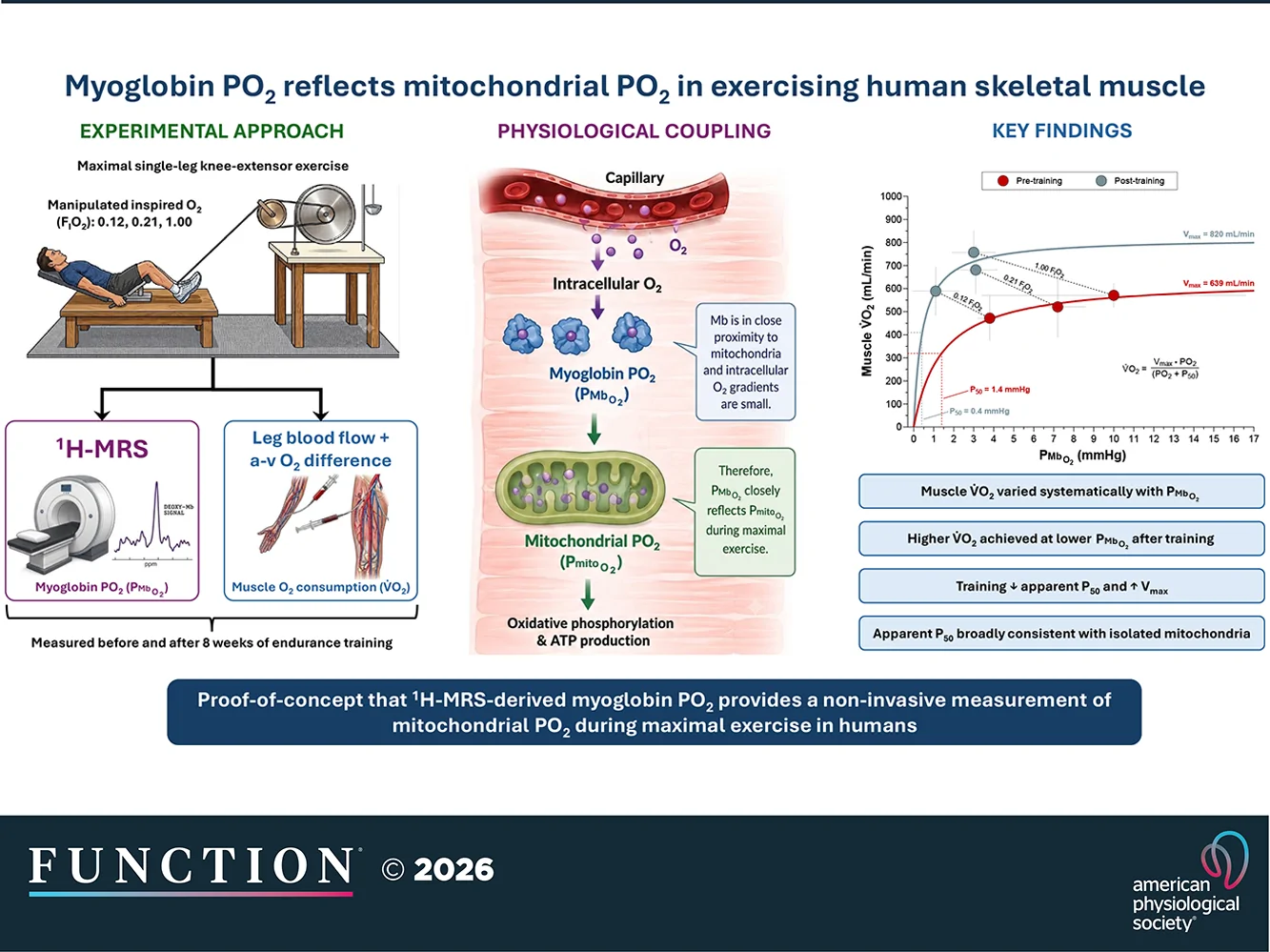

## INTRODUCTION

Skeletal muscle mitochondrial partial pressure of O_2_ (${\mathrm{Pmito}}_{{\text{O}}_{2}}$) is a fundamental determinant of oxidative phosphorylation, metabolic regulation, hypoxia-sensitive signaling, and exercise capacity in health and disease (1, 2). Yet ${\mathrm{Pmito}}_{{\text{O}}_{2}}$ remains difficult to measure directly in vivo in humans. Myoglobin-associated Po_2_ ($P\text{M}{\text{b}}_{{\text{O}}_{2}}$), measured by proton magnetic resonance spectroscopy (^1^H-MRS), provides a potential solution because it reflects skeletal muscle intracellular O_2_ availability. A strong physiological argument for $P\text{M}{\text{b}}_{{\text{O}}_{2}}$ as a measure of ${\mathrm{Pmito}}_{{\text{O}}_{2}}$ is that $P\text{M}{\text{b}}_{{\text{O}}_{2}}$ during maximal exercise lies in the low Po_2_ range expected for ${\mathrm{Pmito}}_{{\text{O}}_{2}}$ in vivo. Human ^1^H-MRS studies have shown that Mb saturation is ∼50%–60% during maximal exercise, corresponding to a $P\text{M}{\text{b}}_{{\text{O}}_{2}}$ of ∼3–5 mmHg when calculated using a Mb P_50_ of 3.2 mmHg (3–7). Because O_2_ diffuses from the intracellular compartment into the mitochondria where it is consumed, ${\mathrm{Pmito}}_{{\text{O}}_{2}}$ would not be expected to exceed $P\text{M}{\text{b}}_{{\text{O}}_{2}}$. Thus, during maximal exercise, $P\text{M}{\text{b}}_{{\text{O}}_{2}}$ likely provides an upper bound for ${\mathrm{Pmito}}_{{\text{O}}_{2}}$, with near-zero as the lower bound. Because $P\text{M}{\text{b}}_{{\text{O}}_{2}}$ itself is only a few mmHg during maximal exercise, the absolute difference between $P\text{M}{\text{b}}_{{\text{O}}_{2}}$ and ${\mathrm{Pmito}}_{{\text{O}}_{2}}$ is likely small.

This bounded-Po_2_ argument is supported by theoretical and experimental evidence indicating that intracellular Po_2_ gradients within skeletal muscle are small. Diffusion theory and experimental measurements estimate the Po_2_ gradient from cytosol to mitochondria to be less than 0.1 mmHg, and the gradient from mitochondrial outer surface to cytochrome c oxidase to be ∼0.01 mmHg, owing to the short diffusion distances and high O_2_ permeability of mitochondrial membranes (8–16). Mb is also located in proximity to mitochondria, within ∼1–2 µm, and its spatial distribution, abundance, and O_2_-binding characteristics facilitate rapid equilibration with the local intracellular Po_2_ environment (9, 13). Consistent with this close coupling, Mb desaturation tracks cytochrome c oxidase reduction (11, 13, 17). Together, these observations support $P\text{M}{\text{b}}_{{\text{O}}_{2}}$ as a physiologically grounded, noninvasive measure of ${\mathrm{Pmito}}_{{\text{O}}_{2}}$ during maximal exercise in humans.

If $P\text{M}{\text{b}}_{{\text{O}}_{2}}$ reflects ${\mathrm{Pmito}}_{{\text{O}}_{2}}$ in vivo, then changes in $P\text{M}{\text{b}}_{{\text{O}}_{2}}$ during maximal exercise should be related to changes in muscle O_2_ consumption (${\dot{\text{V}}\text{O}}_{2}$). However, this relationship must be interpreted within the broader control of mitochondrial respiration. Mitochondrial ${\dot{\text{V}}\text{O}}_{2}$ is governed not only by ${\mathrm{Pmito}}_{{\text{O}}_{2}}$ but also by metabolic signals including adenosine diphosphate (ADP), inorganic phosphate (Pi), and the free energy of adenosine triphosphate (ATP) hydrolysis (18–22). At rest and during submaximal exercise, ${\dot{\text{V}}\text{O}}_{2}$ is primarily constrained by metabolic demand despite relatively high $P\text{M}{\text{b}}_{{\text{O}}_{2}}$ (18–23). During maximal exercise, in contrast, metabolic activation is high, and O_2_ availability often contributes to determining the attainable ${\dot{\text{V}}\text{O}}_{2}$ (24). Therefore, manipulating the fraction of inspired O_2_ (${F\text{I}}_{{\text{O}}_{2}}$) during maximal exercise provides a practical way to vary $P\text{M}{\text{b}}_{{\text{O}}_{2}}$ under conditions of high metabolic activation to determine whether muscle maximal oxygen consumption (${\dot{\text{V}}\text{O}}_{2\text{max}}$) changes in a manner consistent with O_2_-dependent mitochondrial respiration.

Because ${\mathrm{Pmito}}_{{\text{O}}_{2}}$ remains difficult to measure directly in humans, prior studies have largely assessed O_2_-dependent mitochondrial respiration in vitro using high-resolution respirometry in mitochondria isolated from muscle biopsies (25–29). These studies commonly interpret the O_2_ dependence of respiration using a hyperbolic framework, where V_max_ represents maximal ${\dot{\text{V}}\text{O}}_{2}$ at saturating Po_2_ and P_50_ represents the Po_2_ at 50% of V_max_ (2, 25–31). Recently, this framework was applied in vivo by Erol et al. (32), who used ^1^H-MRS of deoxygenated Mb in the gastrocnemius during arterial occlusion of the lower limb at rest to derive both $P\text{M}{\text{b}}_{{\text{O}}_{2}}$ and an Mb-derived estimate of muscle ${\dot{\text{V}}\text{O}}_{2}$, yielding an apparent mitochondrial P50 of ∼0.5 mmHg. This work provides a complementary in vivo estimate of the O_2_ dependence of respiration in human skeletal muscle at rest. It remains unknown, however, whether $P\text{M}{\text{b}}_{{\text{O}}_{2}}$ behaves as expected for ${\mathrm{Pmito}}_{{\text{O}}_{2}}$ during maximal exercise, when metabolic activation is high and O_2_ availability contributes to determining the attainable ${\dot{\text{V}}\text{O}}_{2}$, and when muscle ${\dot{\text{V}}\text{O}}_{2}$ is measured independently of the Mb signal. In the present study, we used this framework as a physiological reference to compare apparent mitochondrial P_50_ estimates derived from the in vivo muscle ${\dot{\text{V}}\text{O}}_{2}$-$P\text{M}{\text{b}}_{{\text{O}}_{2}}$ relationship with prior assessments of the O_2_ dependence of mitochondrial respiration. The purpose of this study was to evaluate $P\text{M}{\text{b}}_{{\text{O}}_{2}}$ as a measure of in vivo skeletal muscle ${\mathrm{Pmito}}_{{\text{O}}_{2}}$ during maximal exercise in humans. We analyzed data from our previous study (24), leveraging corresponding measurements of muscle ${\dot{\text{V}}\text{O}}_{2\text{max}}$ and $P\text{M}{\text{b}}_{{\text{O}}_{2}}$ during maximal exercise across several ${F\text{I}}_{{\text{O}}_{2}}$ conditions before and after endurance exercise training. We hypothesized that $P\text{M}{\text{b}}_{{\text{O}}_{2}}$ during maximal exercise would vary systematically with muscle ${\dot{\text{V}}\text{O}}_{2\text{max}}$ across ${F\text{I}}_{{\text{O}}_{2}}$ conditions and yield apparent mitochondrial P_50_ values consistent with published in vitro mitochondrial respiration studies. This analysis was intended as a data-supported proof-of-concept evaluation of $P\text{M}{\text{b}}_{{\text{O}}_{2}}$ as a measure of ${\mathrm{Pmito}}_{{\text{O}}_{2}}$, rather than as a definitive kinetic characterization of mitochondrial respiratory control.

## METHODS

### Study Design and Participants

This study is a secondary analysis of data from our previous investigation of the determinants of muscle ${\dot{\text{V}}\text{O}}_{2\text{max}}$ before and after endurance exercise training (24), with a novel focus on the relationship between muscle ${\dot{\text{V}}\text{O}}_{2}$ and $P\text{M}{\text{b}}_{{\text{O}}_{2}}$ during maximal single-leg knee-extensor exercise (KE) across varying ${F\text{I}}_{{\text{O}}_{2}}$ conditions. Five initially sedentary males (age: 27 ± 5 yr, height: 176 ± 8 cm, body mass 73 ± 8 kg, cycle ${\dot{\text{V}}\text{O}}_{2\text{max}}$: 35.2 ± 4.9 mL·kg^−1^·min^−1^) without a history of cardiovascular or pulmonary disease completed the study. Ethical approval, participant characteristics, training procedures, and full experimental methods were described in the initial investigation (24).

### Experimental Protocol and Data Analysis

Participants completed study visits in San Diego, CA, to measure muscle ${\dot{\text{V}}\text{O}}_{2\text{max}}$ and study visits in Philadelphia, PA, to measure $P\text{M}{\text{b}}_{{\text{O}}_{2}}$ within a 14-day timespan. During each visit, participants performed maximal KE under three ${F\text{I}}_{{\text{O}}_{2}}$ conditions (0.12, 0.21, and 1.00) in randomized order, both before and after 8 wk of KE endurance training. The exercise protocols consisted of a short, unweighted stage (∼5 min) followed by stepwise increases in work rate every 2–3 min to maximal effort. The same KE ergometer was used at both locations, and the work rates were matched across locations within participants and ${F\text{I}}_{{\text{O}}_{2}}$ conditions, with at least 2 h of rest between trials.

We measured muscle ${\dot{\text{V}}\text{O}}_{2\text{max}}$ using the direct Fick principle from leg blood flow and the arterial-venous O_2_ content difference measured near the end of the maximal exercise stage. In brief, two catheters (radial artery and femoral vein) and a thermocouple (femoral vein) were placed using sterile technique, as previously described (6, 33). We measured leg blood flow using thermodilution and thermal balance principles by infusing iced saline through the femoral venous catheter to decrease blood temperature by ∼1°C (34). The infusion rate and resulting temperature change were used to calculate blood flow. We also sampled arterial and venous blood to measure Po_2_, pH, O_2_ saturation, and hemoglobin (Hb) concentration using a blood gas analyzer and CO-oximeter. Arterial (CaO_2_) and venous (CvO_2_) O_2_ content were calculated as 1.39 × O_2_ saturation × [Hb] + 0.003 × Po_2_, and muscle ${\dot{\text{V}}\text{O}}_{2\text{max}}$ was determined as blood flow × (CaO_2_ − CvO_2_). To measure $P\text{M}{\text{b}}_{{\text{O}}_{2}}$, we used ^1^H-MRS of deoxygenated Mb during matched KE protocols performed in a 2.0-Tesla imaging magnet. A surface coil positioned over the quadriceps was used to acquire proton spectra, and the deoxygenated Mb signal was quantified following standard preprocessing procedures, as described previously (6, 35). Fractional Mb desaturation was normalized to an ischemic signal and converted to $P\text{M}{\text{b}}_{{\text{O}}_{2}}$ using the Mb-O_2_ dissociation relationship (*Eq. 1*), assuming a muscle temperature of 39°C and an Mb P_50_ of 3.2 mmHg (3, 36):

(*1*) $$P_{Mb}O_{2}=MbP_{50}\;\times \frac{S_{Mb}O_{2}}{\left(1-S_{Mb}O_{2}\right)}$$

where S_Mb_O_2_ is fractional Mb saturation.

Assuming that mitochondrial respiration approaches zero as Po_2_ approaches zero, the grouped muscle ${\dot{\text{V}}\text{O}}_{2}$-$P\text{M}{\text{b}}_{{\text{O}}_{2}}$ data were characterized before and after training using a canonical hyperbolic model:

(*2*) $${\dot{\text{V}}\text{O}}_{2}=\frac{{\text{V}}_{\text{max}}\times {P\text{O}}_{2}}{\left({P\text{O}}_{2\;}+P_{50}\right)}$$

where V_max_ represents the asymptotic maximal ${\dot{\text{V}}\text{O}}_{2}$ at saturating Po_2_ and P_50_ represents the Po_2_ at 50% of V_max_. These apparent mitochondrial P_50_ values were compared with previously reported values from in vitro human skeletal muscle-isolated mitochondrial respiration studies (0.3 to 1.3 mmHg) (26–29). To assess how the assumed Mb P_50_ used in the Mb O_2_ saturation-$P\text{M}{\text{b}}_{{\text{O}}_{2}}$ relationship influenced the apparent mitochondrial P_50_ values derived from the muscle ${\dot{\text{V}}\text{O}}_{2}$-$P\text{M}{\text{b}}_{{\text{O}}_{2}}$ relationship, we performed a sensitivity analysis in which the data were reanalyzed using Mb P_50_ values of 1.0, 3.2, and 6.0 mmHg, spanning the range of values reported in the literature. As a complementary, nonmechanistic analysis that did not assume a hyperbolic relationship, we also determined a linear P_50_ for each participant by using the slope defined by the origin and the lowest $P\text{M}{\text{b}}_{{\text{O}}_{2}}$ to calculate the $P\text{M}{\text{b}}_{{\text{O}}_{2}}$ corresponding to 50% of that ${\dot{\text{V}}\text{O}}_{2\text{max}}$ value.

## RESULTS

The muscle ${\dot{\text{V}}\text{O}}_{2\text{max}}$ and $P\text{M}{\text{b}}_{{\text{O}}_{2}}$ responses to ${F\text{I}}_{{\text{O}}_{2}}$ before and after KE endurance training are shown in Fig. 1. Muscle ${\dot{\text{V}}\text{O}}_{2\text{max}}$ increased with ${F\text{I}}_{{\text{O}}_{2}}$ in all participants following training and in the majority before training. In addition, all participants exhibited a decrease in $P\text{M}{\text{b}}_{{\text{O}}_{2}}$ from 0.21 to 0.12 ${F\text{I}}_{{\text{O}}_{2}}$ both before and after training. Consistent with this, muscle ${\dot{\text{V}}\text{O}}_{2\text{max}}$ increased with ${F\text{I}}_{{\text{O}}_{2}}$ following training, whereas $P\text{M}{\text{b}}_{{\text{O}}_{2}}$ was consistently lower after training across all ${F\text{I}}_{{\text{O}}_{2}}$ conditions.

**Figure 1.**
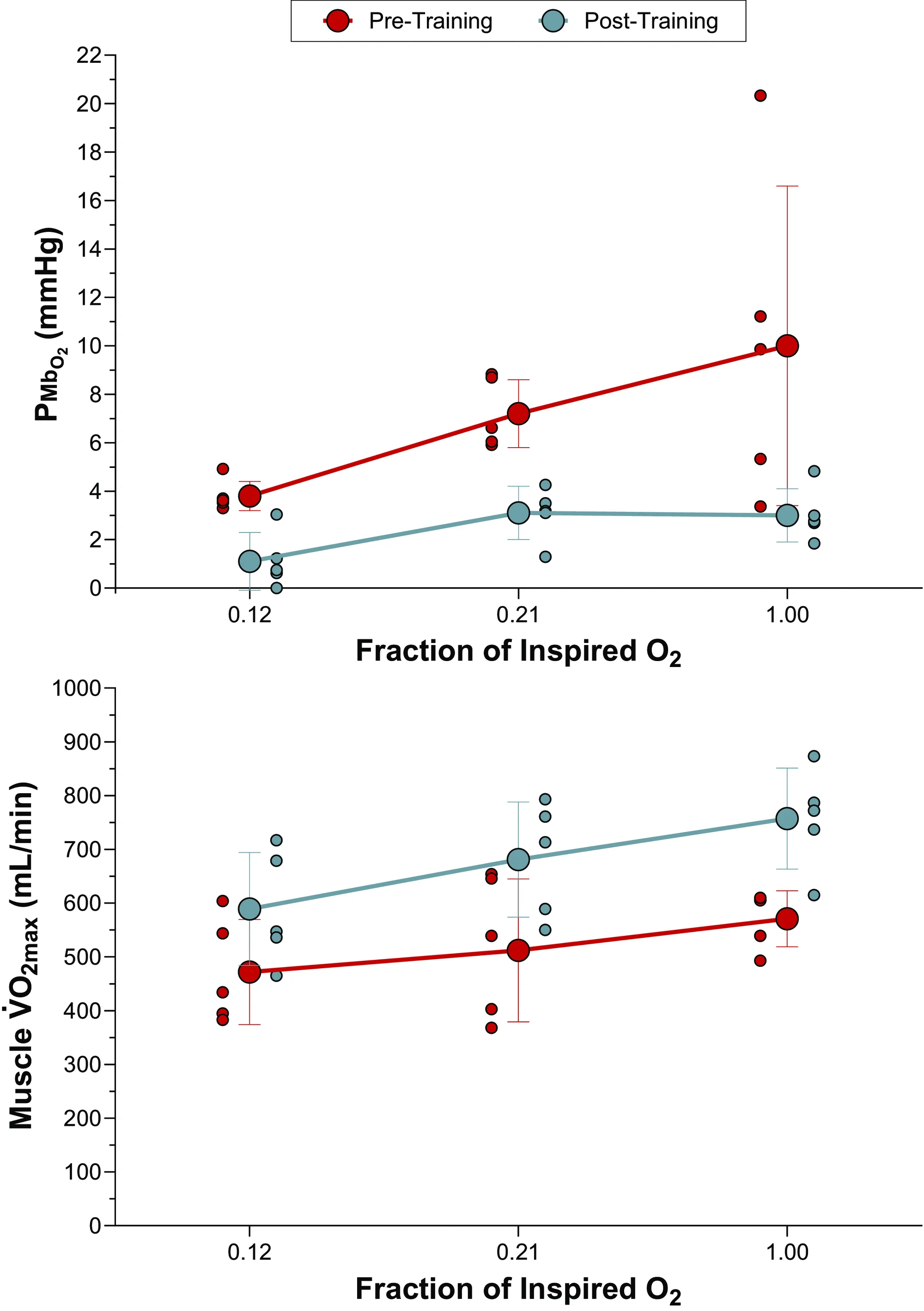
Muscle $P\text{M}{\text{b}}_{{\text{O}}_{2}}$ and ${\dot{\text{V}}\text{O}}_{2\text{max}}$ responses to ${F\text{I}}_{{\text{O}}_{2}}$ before and after endurance training. Muscle myoglobin-associated Po_2_ ($P\text{M}{\text{b}}_{{\text{O}}_{2}}$, *top*) and maximal oxygen uptake (${\dot{\text{V}}\text{O}}_{2\text{max}}$, *bottom*) were measured during maximal knee-extensor exercise (KE) across ${F\text{I}}_{{\text{O}}_{2}}$ conditions of 0.12, 0.21, and 1.00 before and after 8 wk of KE training. Data are from *n* = 5 participants. Small symbols represent individual participant values, and large symbols with error bars represent means ± SD. Across ${F\text{I}}_{{\text{O}}_{2}}$ conditions, endurance training increased muscle ${\dot{\text{V}}\text{O}}_{2\text{max}}$ and decreased $P\text{M}{\text{b}}_{{\text{O}}_{2}}$, demonstrating that greater O_2_ utilization was achieved at lower intracellular Po_2_ after training. ${F\text{I}}_{{\text{O}}_{2}}$, fractions of inspired oxygen; $P\text{M}{\text{b}}_{{\text{O}}_{2}}$, myoglobin-associated partial pressure of oxygen; ${\dot{\text{V}}\text{O}}_{2\text{max}}$, maximal oxygen consumption.

The hyperbolic models for the muscle ${\dot{\text{V}}\text{O}}_{2}$-$P\text{M}{\text{b}}_{{\text{O}}_{2}}$ relationships are illustrated in Fig. 2. Before training, $P\text{M}{\text{b}}_{{\text{O}}_{2}}$ values ranged from 3.8 to 10.0 mmHg, whereas posttraining values ranged from 1.1 to 3.1 mmHg. When interpreted within a hyperbolic framework, the ${\dot{\text{V}}\text{O}}_{2}$-$P\text{M}{\text{b}}_{{\text{O}}_{2}}$ relationships were consistent with the expected O_2_ dependence of mitochondrial respiration, with KE endurance training shifting the relationship upward and leftward. Pretraining, the apparent mitochondrial P_50_ was 1.4 mmHg and V_max_ was 639 mL/min, whereas posttraining the apparent mitochondrial P_50_ was 0.4 mmHg and V_max_ was 820 mL/min. These apparent mitochondrial P_50_ values are shown alongside P_50_ values previously reported for human skeletal muscle-isolated mitochondria studied in vitro in Fig. 3. The posttraining apparent mitochondrial P_50_ value fell within the reported range of 0.3 to 1.3 mmHg, whereas the pretraining value was slightly above this range. The sensitivity analysis examining the influence of the assumed Mb P_50_ on the derived muscle ${\dot{\text{V}}\text{O}}_{2}$-$P\text{M}{\text{b}}_{{\text{O}}_{2}}$ apparent mitochondrial P_50_ values is shown in Fig. 4. Across assumed Mb P_50_ values of 1.0, 3.2, and 6.0 mmHg, the apparent mitochondrial P_50_ values scaled proportionally, with pretraining estimates ranging from 0.44 to 2.64 mmHg and posttraining estimates ranging from 0.13 to 0.80 mmHg. The linear P_50_ values (mean ± SD) were 1.9 ± 0.3 mmHg pretraining and 0.6 ± 0.6 mmHg posttraining.

**Figure 2.**
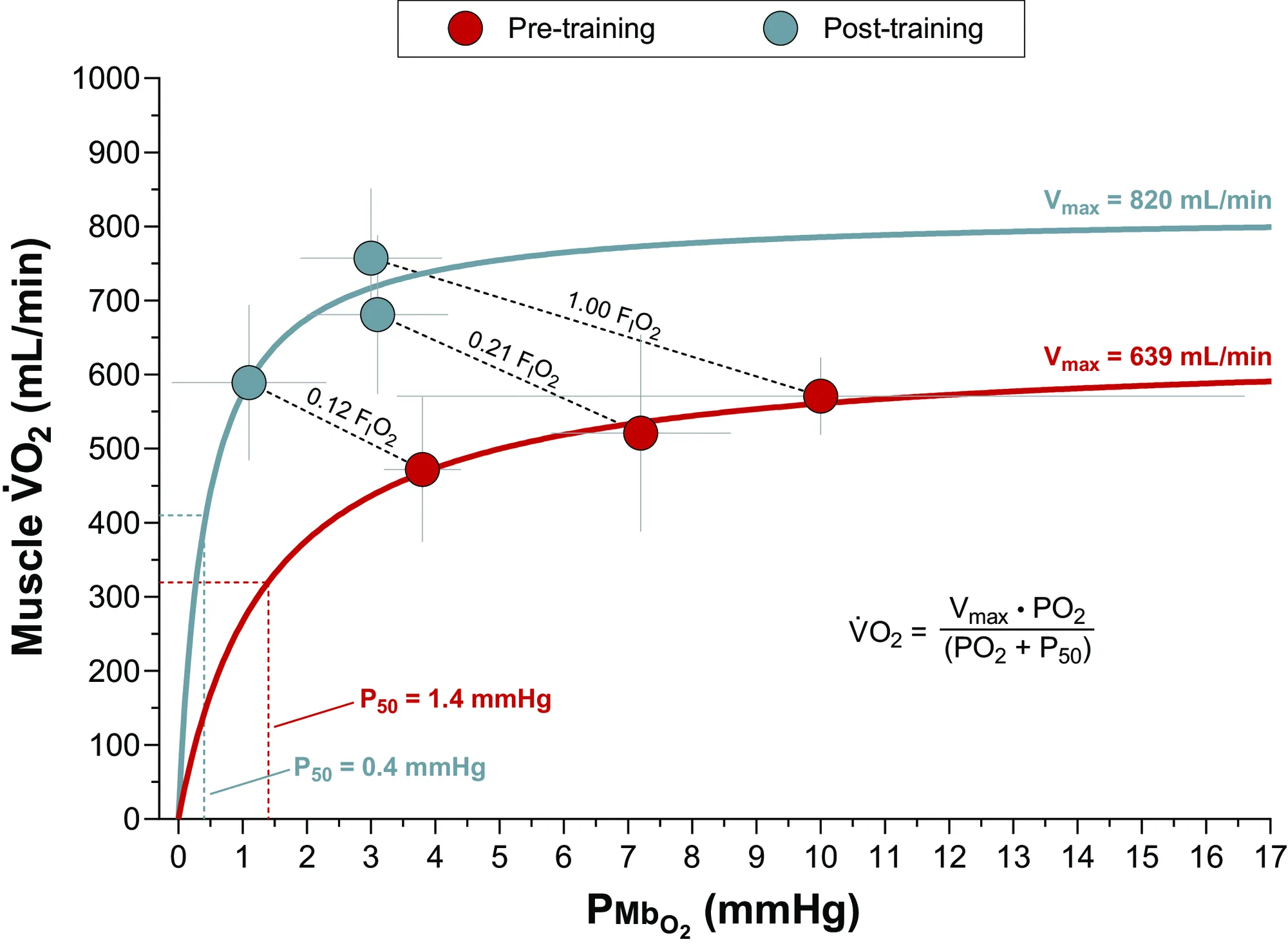
Muscle ${\dot{\text{V}}\text{O}}_{2}$ and $P\text{M}{\text{b}}_{{\text{O}}_{2}}$ relationship during maximal exercise before and after endurance training. Muscle oxygen uptake (${\dot{\text{V}}\text{O}}_{2}$) and myoglobin-associated Po_2_ ($P\text{M}{\text{b}}_{{\text{O}}_{2}}$) were measured during maximal knee-extensor exercise (KE) at ${F\text{I}}_{{\text{O}}_{2}}$ conditions of 0.12, 0.21, and 1.00 before and after 8 wk of KE training. Symbols represent group means for *n* = 5 participants, with error bars indicating between-participant SD. Each symbol represents the condition-specific muscle ${\dot{\text{V}}\text{O}}_{2\text{max}}$, determined by the intersection of the mitochondrial ${\dot{\text{V}}\text{O}}_{2}$-$P\text{M}{\text{b}}_{{\text{O}}_{2}}$ relationship and the prevailing O_2_ transport relationship (37). Curves represent canonical hyperbolic fits to the grouped ${\dot{\text{V}}\text{O}}_{2}$-$P\text{M}{\text{b}}_{{\text{O}}_{2}}$ data before and after training, constrained by the physiological assumption that ${\dot{\text{V}}\text{O}}_{2}$ approaches zero as Po_2_ approaches zero. These fits were used to estimate apparent mitochondrial P_50_ and V_max_ values for comparison with previously published mitochondrial respiration data, rather than as a definitive kinetic characterization of mitochondrial respiration at the individual level. ${F\text{l}}_{{\text{O}}_{2}}$, fractions of inspired oxygen; ${\dot{\text{V}}\text{O}}_{2\text{max}}$, maximal oxygen consumption.

## DISCUSSION

### Principal Findings

The principal and novel finding of this study was that $P\text{M}{\text{b}}_{{\text{O}}_{2}}$, measured noninvasively by ^1^H-MRS, behaved as expected for a measure of in vivo ${\mathrm{Pmito}}_{{\text{O}}_{2}}$ during maximal exercise in humans. Across ${F\text{I}}_{{\text{O}}_{2}}$ conditions, muscle ${\dot{\text{V}}\text{O}}_{2\text{max}}$ varied systematically with $P\text{M}{\text{b}}_{{\text{O}}_{2}}$ before and after endurance exercise training. After training, a higher muscle ${\dot{\text{V}}\text{O}}_{2\text{max}}$ was achieved at a lower $P\text{M}{\text{b}}_{{\text{O}}_{2}}$, consistent with greater O_2_ sensitivity of mitochondrial respiration. This observation is important because it arises directly from the measured data and does not depend on a specific curve-fitting model. When the ${\dot{\text{V}}\text{O}}_{2}$-$P\text{M}{\text{b}}_{{\text{O}}_{2}}$ data were interpreted within a canonical hyperbolic framework, the apparent mitochondrial P_50_ values were low and broadly consistent with values reported for human skeletal muscle-isolated mitochondria studied in vitro. A complementary linear P_50_ analysis, which did not rely on hyperbolic curve fitting, supported the same overall interpretation. Together, these findings provide data-supported, proof-of-concept evidence that $P\text{M}{\text{b}}_{{\text{O}}_{2}}$ reflects ${\mathrm{Pmito}}_{{\text{O}}_{2}}$ during maximal exercise in humans.

### Biological Plausibility

The interpretation that skeletal muscle $P\text{M}{\text{b}}_{{\text{O}}_{2}}$ reflects ${\mathrm{Pmito}}_{{\text{O}}_{2}}$ is biologically plausible for three reasons. First, $P\text{M}{\text{b}}_{{\text{O}}_{2}}$ is low during maximal exercise and in the range expected for ${\mathrm{Pmito}}_{{\text{O}}_{2}}$ in vivo. Because O_2_ diffuses from the intracellular compartment into the mitochondria where it is consumed, ${\mathrm{Pmito}}_{{\text{O}}_{2}}$ would not be expected to exceed $P\text{M}{\text{b}}_{{\text{O}}_{2}}$. Thus, $P\text{M}{\text{b}}_{{\text{O}}_{2}}$ provides an upper bound for ${\mathrm{Pmito}}_{{\text{O}}_{2}}$ during maximal exercise. Since the lower bound is near zero and the group mean $P\text{M}{\text{b}}_{{\text{O}}_{2}}$ values across the ${F\text{I}}_{{\text{O}}_{2}}$ conditions were ∼1–10 mmHg (Figs. 1 and 2), the absolute difference between $P\text{M}{\text{b}}_{{\text{O}}_{2}}$ and ${\mathrm{Pmito}}_{{\text{O}}_{2}}$ is likely small (3–7). Second, diffusion theory and experimental evidence indicate that intracellular Po_2_ gradients within skeletal muscle are small (9). Early cryomicrospectrophotometry work was consistent with this interpretation, although the relatively large sampling volume of this technique limits its spatial resolution and ability to resolve subcellular Po_2_ gradients (38). Although there is a substantial Po_2_ gradient, typically >30 mmHg, from capillary blood to the myofiber interior during maximal exercise, intracellular gradients are comparatively small and have been estimated to be less than 0.1 mmHg from cytosol to mitochondria (6, 8–14, 24). This difference arises because mitochondrial surface area greatly exceeds capillary surface area, producing low resistance to O_2_ transport within the myofiber. As a result, the same O_2_ flux produces substantially smaller Po_2_ gradients from the cytosol to the outer mitochondrial membrane than from capillary blood to the myofiber interior (14, 39, 40). The Po_2_ gradient from the mitochondrial outer membrane to cytochrome c oxidase is even smaller, ∼0.01 mmHg, because of the high O_2_ permeability of mitochondrial membranes and short diffusion distances (12, 13, 15, 16). Contemporary capillary function models also emphasize that intramuscular O_2_ transport depends on the extracellular space as a conduit for O_2_ transport in addition to intracellular diffusion (41–43). Third, Mb is well positioned to reflect the local intracellular O_2_ environment relevant to mitochondria. Mb lies near mitochondria, ∼1–2 µm, and its spatial distribution, abundance, and O_2_-binding characteristics facilitate rapid equilibration with intracellular Po_2_ (9, 13). Consistent with this close coupling, Mb desaturation tracks cytochrome c oxidase reduction, supporting a tight relationship between $P\text{M}{\text{b}}_{{\text{O}}_{2}}$ and ${\mathrm{Pmito}}_{{\text{O}}_{2}}$ (11, 13, 17).

It is also important to distinguish O_2_ availability from metabolic activation coupled to metabolic demand. Mitochondrial ${\dot{\text{V}}\text{O}}_{2}$ in vivo is governed not only by ${\mathrm{Pmito}}_{{\text{O}}_{2}}$ but also by metabolic signals such as ADP, Pi, and the free energy of ATP hydrolysis (18–22). At rest and during submaximal exercise, mitochondrial ${\dot{\text{V}}\text{O}}_{2}$ is primarily constrained by metabolic demand despite relatively high $P\text{M}{\text{b}}_{{\text{O}}_{2}}$ values (6, 23). During maximal exercise, in contrast, metabolic activation is high and O_2_ availability can contribute to determining the attainable ${\dot{\text{V}}\text{O}}_{2}$. This distinction helps reconcile the present findings with prior observations that $P\text{M}{\text{b}}_{{\text{O}}_{2}}$ does not decline linearly with increasing work rate but instead plateaus beyond ∼50%–60% of maximal exercise (5, 6). In that setting, ${\dot{\text{V}}\text{O}}_{2}$ continues to increase with work rate despite a relatively constant $P\text{M}{\text{b}}_{{\text{O}}_{2}}$ because metabolic activation and O_2_ transport increase proportionally. This distinction is also apparent when the present findings are considered alongside resting measurements. Erol et al. (32) reported that the resting muscle ${\dot{\text{V}}\text{O}}_{2}$-$P\text{M}{\text{b}}_{{\text{O}}_{2}}$ relationship saturates at the basal metabolic rate, well above the Po_2_ range over which respiration becomes O_2_-dependent, such that resting ${\dot{\text{V}}\text{O}}_{2}$ is constrained by metabolic demand rather than by O_2_ availability. During maximal exercise, the effective mitochondrial respiratory capacity is markedly higher and $P\text{M}{\text{b}}_{{\text{O}}_{2}}$ falls into the range over which O_2_ availability can contribute to determining the attainable ${\dot{\text{V}}\text{O}}_{2}$. In the present study, we manipulated ${F\text{I}}_{{\text{O}}_{2}}$ during maximal exercise to vary $P\text{M}{\text{b}}_{{\text{O}}_{2}}$ while maintaining high metabolic activation. Thus, graded exercise and ${F\text{I}}_{{\text{O}}_{2}}$ manipulation during maximal exercise are not mechanistically equivalent interventions. Graded exercise increases metabolic demand and thereby changes metabolic activation, effective mitochondrial respiratory capacity, and O_2_ transport. In contrast, ${F\text{I}}_{{\text{O}}_{2}}$ manipulation during maximal exercise primarily alters $P\text{M}{\text{b}}_{{\text{O}}_{2}}$, allowing ${\dot{\text{V}}\text{O}}_{2\text{max}}$ to vary along a similarly expressed maximal respiratory framework. Ultimately, intracellular Po_2_ in skeletal muscle reflects the integrated balance between metabolic activation and O_2_ transport. Metabolic activation alters the effective mitochondrial respiratory capacity, and O_2_ transport is determined by convective and diffusive function. Together, this framework supports the biological plausibility of $P\text{M}{\text{b}}_{{\text{O}}_{2}}$ reflecting ${\mathrm{Pmito}}_{{\text{O}}_{2}}$ in skeletal muscle.

### Physiological Validity

A key indicator of the physiological validity of $P\text{M}{\text{b}}_{{\text{O}}_{2}}$ reflecting ${\mathrm{Pmito}}_{{\text{O}}_{2}}$ in skeletal muscle is that the measured responses to ${F\text{I}}_{{\text{O}}_{2}}$ and endurance training were internally consistent with expected muscle physiology. Endurance training increased muscle ${\dot{\text{V}}\text{O}}_{2\text{max}}$ and decreased $P\text{M}{\text{b}}_{{\text{O}}_{2}}$ across ${F\text{I}}_{{\text{O}}_{2}}$ conditions (Figs. 1 and 2). Thus, after training, the muscle consumed more O_2_ while operating at a lower intracellular Po_2_. This model-independent observation supports the interpretation that intracellular O_2_ availability contributes to determining mitochondrial respiration during maximal exercise. The ${\dot{\text{V}}\text{O}}_{2}$-$P\text{M}{\text{b}}_{{\text{O}}_{2}}$ relationship also behaved as expected when interpreted within a canonical hyperbolic framework of mitochondrial O_2_ dependence (Fig. 2). Before and after endurance training, the apparent mitochondrial P_50_ values derived from the hyperbolic analysis were low and broadly consistent with values reported for human skeletal muscle-isolated mitochondria studied in vitro (Fig. 3). We focused on in vitro P_50_ values measured during complex I + II state III respiration in human vastus lateralis isolated mitochondria because these conditions provide the closest available comparison with maximal exercise in vivo (26–29). However, we recognize that isolated mitochondrial preparations differ from intact exercise muscle with respect to substrate availability, energetic state, and metabolic regulation. A similar comparison cannot be made for V_max_ data because in vitro measurements reflect isolated mitochondrial mass, whereas the present data reflect whole muscle. The present apparent mitochondrial P_50_ values can also be considered alongside a recent in vivo estimate of mitochondrial O_2_ affinity in human skeletal muscle. Using ^1^H-MRS of Mb desaturation of the gastrocnemius during arterial occlusion of the resting lower limb, Erol et al. (32) reported an apparent mitochondrial P_50_ of ∼0.5 mmHg, within the same low Po_2_ range as the present estimates. This consistency is notable given the substantial differences between the two approaches. Erol et al. (32) studied resting muscle during ischemia and derived both $P\text{M}{\text{b}}_{{\text{O}}_{2}}$ and an estimate of muscle ${\dot{\text{V}}\text{O}}_{2}$ from the same Mb-desaturation time course, whereas the present study examined maximal exercise, manipulated $P\text{M}{\text{b}}_{{\text{O}}_{2}}$ across ${F\text{I}}_{{\text{O}}_{2}}$ conditions, and measured muscle ${\dot{\text{V}}\text{O}}_{2\text{max}}$ independently of the Mb signal. The convergence of two methodologically distinct in vivo approaches on similarly low apparent mitochondrial P_50_ values strengthens the interpretation that $P\text{M}{\text{b}}_{{\text{O}}_{2}}$ lies within the Po_2_ range relevant to mitochondrial respiration in vivo. The linear P_50_ analysis supports the same interpretation without assuming a specific form of the muscle ${\dot{\text{V}}\text{O}}_{2}$-$P\text{M}{\text{b}}_{{\text{O}}_{2}}$ relationship. Although the linear P_50_ values were slightly higher than those derived from the hyperbolic model, both approaches indicated that $P\text{M}{\text{b}}_{{\text{O}}_{2}}$ during maximal exercise lies in the low Po_2_ range expected for ${\mathrm{Pmito}}_{{\text{O}}_{2}}$ in vivo. In addition, this interpretation is further supported by the absolute $P\text{M}{\text{b}}_{{\text{O}}_{2}}$ values measured during maximal exercise. Across the present ${F\text{I}}_{{\text{O}}_{2}}$ conditions, group mean $P\text{M}{\text{b}}_{{\text{O}}_{2}}$ values ranged from ∼1 to 10 mmHg (Figs. 1 and 2). ${\mathrm{Pmito}}_{{\text{O}}_{2}}$ would not be expected to exceed $P\text{M}{\text{b}}_{{\text{O}}_{2}}$ because O_2_ diffuses from the intracellular compartment into the mitochondria where it is consumed. Thus, $P\text{M}{\text{b}}_{{\text{O}}_{2}}$ likely provides an upper bound for ${\mathrm{Pmito}}_{{\text{O}}_{2}}$ during maximal exercise, with near-zero as the lower bound and the observed $P\text{M}{\text{b}}_{{\text{O}}_{2}}$ values defining the upper bound. This narrow physiological range supports $P\text{M}{\text{b}}_{{\text{O}}_{2}}$ as a close approximation of ${\mathrm{Pmito}}_{{\text{O}}_{2}}$ during maximal exercise.

**Figure 3.**
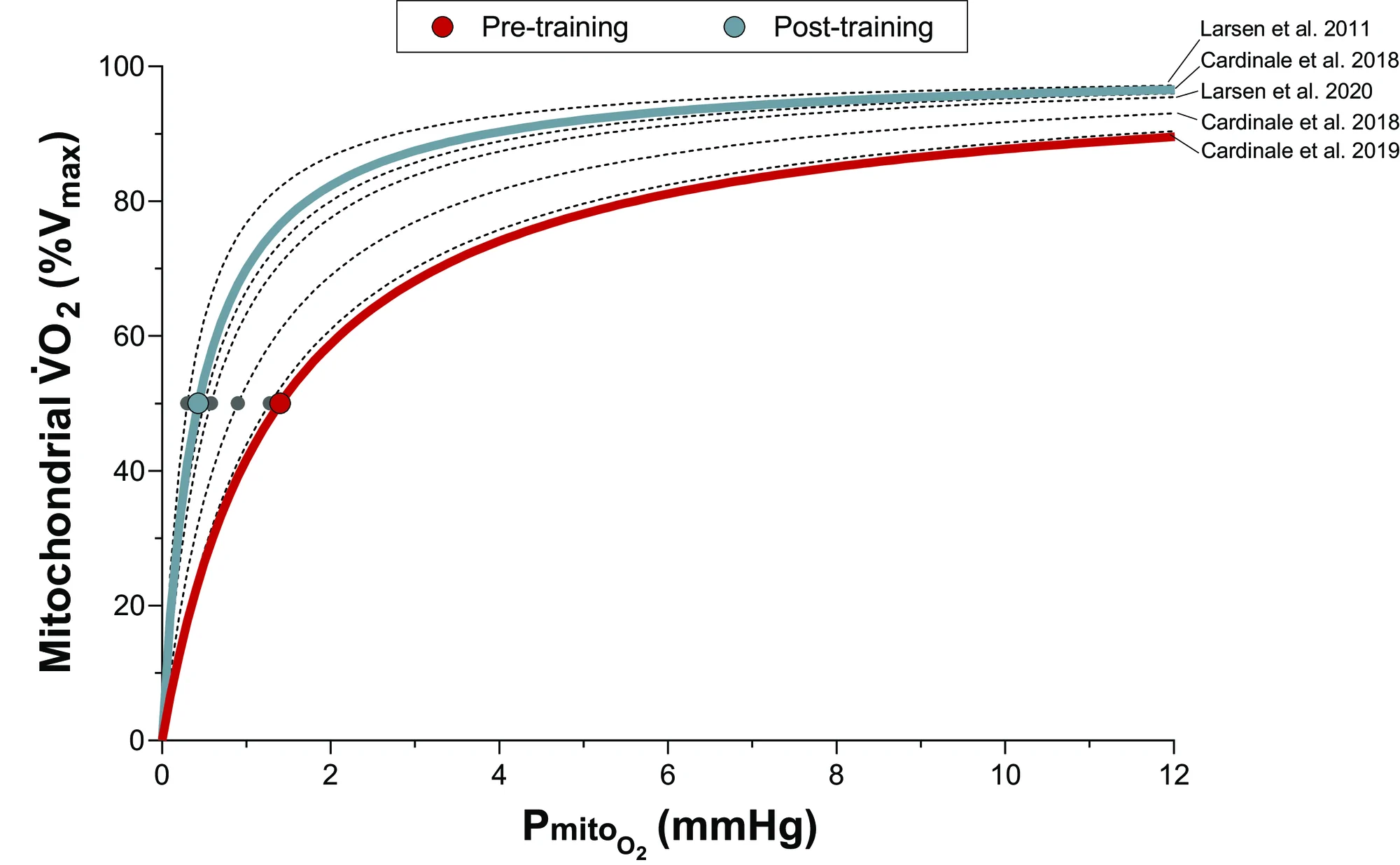
Apparent mitochondrial P_50_ values relative to published in vitro skeletal muscle-isolated mitochondria values. The current in vivo muscle oxygen uptake (${\dot{\text{V}}\text{O}}_{2}$) and myoglobin-associated Po_2_ ($P\text{M}{\text{b}}_{{\text{O}}_{2}}$) relationships for *n* = 5 participants are shown together with previously published O_2_-dependent respiration relationships from in vitro human skeletal muscle-isolated mitochondria (26–29). For comparison across preparations, ${\dot{\text{V}}\text{O}}_{2}$ values are normalized to their respective V_max_ values, and $P\text{M}{\text{b}}_{{\text{O}}_{2}}$ from the present study is interpreted as $\text{Pmit}{\text{o}}_{{\text{O}}_{2}}$. Filled circles indicate the apparent mitochondrial P_50_ values derived from the present in vivo data and the P_50_ values reported from the in vitro mitochondrial studies. This comparison is intended to provide physiological context for the apparent O_2_ sensitivity of the in vivo ${\dot{\text{V}}\text{O}}_{2}$-$P\text{M}{\text{b}}_{{\text{O}}_{2}}$ relationships, not to imply direct equivalence between in vivo whole muscle and in vitro isolated mitochondrial preparations. $\text{Pmit}{\text{o}}_{{\text{O}}_{2}}$, mitochondrial partial pressure of oxygen.

The training-induced shift in the ${\dot{\text{V}}\text{O}}_{2}$-$P\text{M}{\text{b}}_{{\text{O}}_{2}}$ relationship is also consistent physiologically. The decrease in the apparent mitochondrial P_50_ is consistent with greater O_2_ sensitivity of mitochondrial respiration, whereas the increase in the V_max_ is consistent with greater oxidative phosphorylation capacity (28, 44). In the untrained state, $P\text{M}{\text{b}}_{{\text{O}}_{2}}$ during maximal exercise was higher, indicating that the muscle was operating closer to the plateau region of the mitochondrial respiration curve, where ${\dot{\text{V}}\text{O}}_{2}$ is less sensitive to further increases in $P\text{M}{\text{b}}_{{\text{O}}_{2}}$. After training, $P\text{M}{\text{b}}_{{\text{O}}_{2}}$ during maximal exercise was lower, indicating that the muscle was now operating closer to the steeper portion of the curve, where changes in $P\text{M}{\text{b}}_{{\text{O}}_{2}}$ would be expected to have a greater influence on muscle ${\dot{\text{V}}\text{O}}_{2}$. Importantly, muscle ${\dot{\text{V}}\text{O}}_{2\text{max}}$ is determined by the interaction between O_2_ transport and mitochondrial respiration, rather than either system in isolation (37). Accordingly, adaptations in both mitochondrial function and O_2_ transport capacity act together to determine the response of ${\dot{\text{V}}\text{O}}_{2\text{max}}$ to endurance training in vivo.

### Sensitivity of the Apparent Mitochondrial P_50_ to the Assumed Mb P_50_

The apparent mitochondrial P_50_ values depend directly on the Mb P_50_ used to convert Mb saturation to $P\text{M}{\text{b}}_{{\text{O}}_{2}}$. Reported Mb P_50_ values span ∼1–6 mmHg across human and animal studies (3, 13). In the present study, as in our previous work, we used a Mb P_50_ of 3.2 mmHg because this value was determined from human Mb and is appropriate for an estimated muscle temperature of ∼39°C during maximal exercise (3, 36). Based on the Mb-O_2_ dissociation curve, changing the assumed Mb P_50_ proportionally rescales all $P\text{M}{\text{b}}_{{\text{O}}_{2}}$ values. Because $P\text{M}{\text{b}}_{{\text{O}}_{2}}$ is the independent variable in the muscle ${\dot{\text{V}}\text{O}}_{2}$-$P\text{M}{\text{b}}_{{\text{O}}_{2}}$ relationship, the apparent mitochondrial P_50_ scales proportionally with the assumed Mb P_50_ (Fig. 4). Thus, if the true Mb P_50_ differs from 3.2 mmHg, the absolute apparent mitochondrial P_50_ values would shift proportionally, whereas the relative differences across ${F\text{I}}_{{\text{O}}_{2}}$ conditions and between pre- and posttraining would be preserved. This proportional dependence is important when comparing the present apparent mitochondrial P_50_ values with published in vitro mitochondrial P_50_ values (26–29). This consideration is also important when comparing the present results with the resting estimate of Erol et al. (32), who used an Mb P_50_ of 2.2 mmHg based on an estimated resting muscle temperature of ∼36°C. For comparison on the same assumed Mb P_50_ scale, their apparent mitochondrial P_50_ of ∼0.5 mmHg would scale to ∼0.7 mmHg using the Mb P_50_ of 3.2 mmHg applied in the present study. Uncertainty in the assumed Mb P_50_ can influence the absolute magnitude of the apparent mitochondrial P_50_ values and their agreement with the in vitro range. However, this uncertainty does not alter the primary finding that the ${\dot{\text{V}}\text{O}}_{2}$-$P\text{M}{\text{b}}_{{\text{O}}_{2}}$ relationship is consistent with O_2_-dependent mitochondrial respiration under conditions of high metabolic activation. Moreover, the linear P_50_ analysis supports the same conclusion without depending on hyperbolic model fitting.

**Figure 4.**
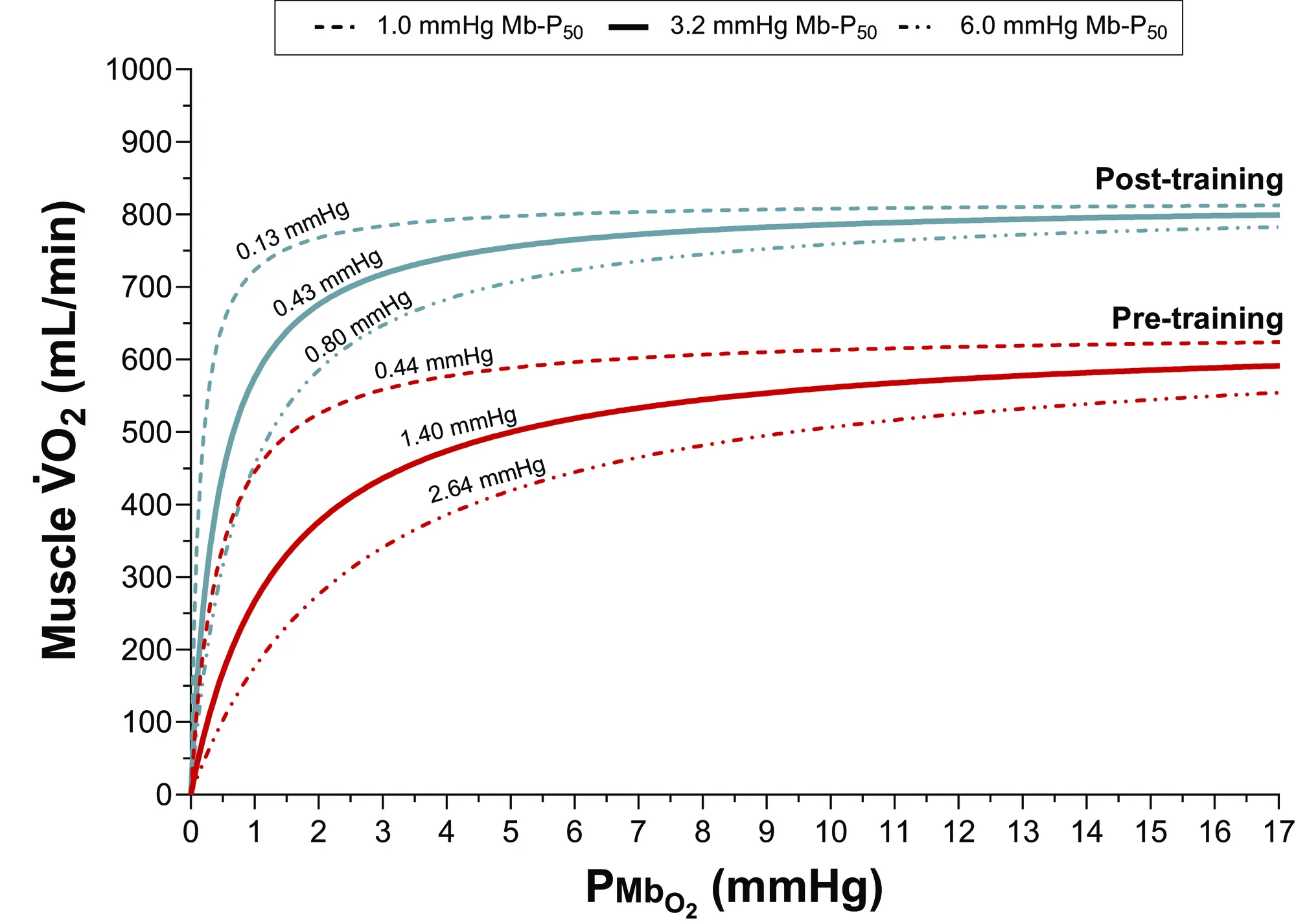
Sensitivity of the apparent mitochondrial P_50_ values to the assumed Mb P_50_. The grouped in vivo muscle oxygen uptake (${\dot{\text{V}}\text{O}}_{2}$) and myoglobin-associated Po_2_ ($P\text{M}{\text{b}}_{{\text{O}}_{2}}$) data from *n* = 5 participants were reanalyzed using Mb P_50_ values of 1.0, 3.2, and 6.0 mmHg to illustrate how the Mb-O_2_ dissociation curve P_50_ influences the derived in vivo ${\dot{\text{V}}\text{O}}_{2}$-$P\text{M}{\text{b}}_{{\text{O}}_{2}}$ relationships and apparent mitochondrial P_50_ values. Curves represent canonical hyperbolic fits to the grouped data before and after training under each assumed Mb P_50_ value. The corresponding apparent mitochondrial P_50_ values are indicated for each fit. This sensitivity analysis illustrates that changes in the assumed Mb P_50_ proportionally rescale $P\text{M}{\text{b}}_{{\text{O}}_{2}}$ and the derived apparent mitochondrial P_50_ values.

### Clinical and Research Implications

The ability to noninvasively assess ${\mathrm{Pmito}}_{{\text{O}}_{2}}$ in vivo has important physiological and clinical implications. Quantifying ${\mathrm{Pmito}}_{{\text{O}}_{2}}$ may improve understanding of hypoxia-sensitive gene expression and oxidative stress, particularly pathways regulated by hypoxia-inducible factor 1 (HIF-1) that respond to intracellular O_2_ availability (1, 2). In addition, this approach may enable assessment of spatial heterogeneity in mitochondrial O_2_ supply-utilization matching by sampling different muscles with ^1^H-MRS. This capability may be particularly important in conditions such as cardiovascular disease, peripheral artery disease, or chronic obstructive pulmonary disease, where impaired matching may lead to severe localized intracellular hypoxia during exercise. This approach may also provide an opportunity to evaluate how muscle fiber-type differences in mitochondrial and O_2_ transport properties influence ${\mathrm{Pmito}}_{{\text{O}}_{2}}$ across muscles, individuals, and training states (45–47). Knowledge of ${\mathrm{Pmito}}_{{\text{O}}_{2}}$ may also improve estimates of muscle diffusional O_2_ conductance (D_M_O_2_), calculated as muscle ${\dot{\text{V}}\text{O}}_{2}$ divided by the difference between mean capillary Po_2_ and ${\mathrm{Pmito}}_{{\text{O}}_{2}}$ (37, 48, 49). D_M_O_2_ is an important determinant of muscle ${\dot{\text{V}}\text{O}}_{2\text{max}}$ and varies across populations and interventions (24, 50–52). Incorporating ${\mathrm{Pmito}}_{{\text{O}}_{2}}$ would remove a major source of uncertainty in these calculations and may allow for more accurate assessment of D_M_O_2_ across submaximal exercise intensities. Another implication is that this approach provides a measure of in vivo mitochondrial function that complements, but extends beyond, near-infrared spectroscopy (NIRS). Although NIRS can assess muscle oxygenation, it cannot distinguish Mb from Hb contributions, the Hb signal reflects an uncertain mix of arterial, microvascular, and venous blood, and derived concentrations may be markedly lower than biochemically determined Mb concentrations. In contrast, ^1^H-MRS specifically measures Mb desaturation, providing a more direct assessment of intracellular oxygenation. Overall, the ability to measure in vivo ${\mathrm{Pmito}}_{{\text{O}}_{2}}$ opens new opportunities to investigate the mechanistic role of O_2_ availability in gene regulation, muscle adaptation, and physiological function in health and disease.

### Experimental Considerations

Several experimental considerations should be acknowledged. Muscle ${\dot{\text{V}}\text{O}}_{2\text{max}}$ and $P\text{M}{\text{b}}_{{\text{O}}_{2}}$ were not measured simultaneously but were obtained at separate locations within a 14-day period using the same KE ergometer, with work rates matched within participants across locations and ${F\text{I}}_{{\text{O}}_{2}}$ conditions. Consequently, each ${\dot{\text{V}}\text{O}}_{2\text{max}}$-$P\text{M}{\text{b}}_{{\text{O}}_{2}}$ pair reflects two matched exercise bouts rather than one. Day-to-day biological variability may have influenced these measurements, but such variability would not be expected to act systematically in one direction, and the responses to ${F\text{I}}_{{\text{O}}_{2}}$ and training were consistent within participants. Nonetheless, simultaneous measurement of muscle ${\dot{\text{V}}\text{O}}_{2\text{max}}$ and $P\text{M}{\text{b}}_{{\text{O}}_{2}}$ would strengthen characterization of the ${\dot{\text{V}}\text{O}}_{2}$-$P\text{M}{\text{b}}_{{\text{O}}_{2}}$ relationship and should be a goal of future work. An additional consideration is that arterial blood was sampled from the radial artery, whereas venous blood was sampled from the femoral vein. Thus, calculation of the arterial-venous O_2_ content difference assumes that systemic arterial O_2_ content is representative of arterial inflow to the exercising leg. This assumption is well founded physiologically because arterial O_2_ content, established by pulmonary gas exchange and the O_2_-carrying characteristics of the blood, remains essentially uniform throughout the systemic arterial circulation before tissue-level O_2_ extraction occurs. In contrast, venous O_2_ content varies among tissues according to local O_2_ delivery and utilization, and blood flow and venous O_2_ content were therefore measured in the exercising leg. Accordingly, radial artery blood provides a valid estimate of the O_2_ content of blood entering the exercising limb, and this well-established approach is unlikely to have substantially influenced the present findings. We examined only three ${F\text{I}}_{{\text{O}}_{2}}$ conditions, which may limit the precision of apparent mitochondrial P_50_ estimation. In addition, $P\text{M}{\text{b}}_{{\text{O}}_{2}}$ was measured only during maximal exercise. This approach is appropriate for evaluating the ${\dot{\text{V}}\text{O}}_{2}$-$P\text{M}{\text{b}}_{{\text{O}}_{2}}$ relationship under conditions of high metabolic activation, but it may not extend directly to submaximal exercise intensities, where the effective mitochondrial respiratory capacity is submaximal. We also performed the primary hyperbolic analysis using group-averaged data from a relatively small sample of young healthy males. Future studies should evaluate larger and more diverse populations and examine individual variation in the ${\dot{\text{V}}\text{O}}_{2}$-$P\text{M}{\text{b}}_{{\text{O}}_{2}}$ relationship. Another consideration is that $P\text{M}{\text{b}}_{{\text{O}}_{2}}$ represents an average across the sampled muscle volume and may not capture fine-scale spatial heterogeneity. This averaging may limit the ability to resolve regional or fiber-type-specific differences in intracellular Po_2_ within the same muscle. However, for many physiological and clinical applications, a volume-averaged intracellular Po_2_ may be a more relevant measure of whole muscle function. Moreover, estimation of $P\text{M}{\text{b}}_{{\text{O}}_{2}}$ from Mb saturation is less sensitive at higher oxygenation levels because of the nonlinear Mb O_2_ saturation-$P\text{M}{\text{b}}_{{\text{O}}_{2}}$ relationship. As $P\text{M}{\text{b}}_{{\text{O}}_{2}}$ increases, the dissociation curve flattens, so small differences in Mb saturation produce larger differences in the calculated $P\text{M}{\text{b}}_{{\text{O}}_{2}}$. This reflects a fundamental property of the O_2_ dissociation relationship rather than a limitation of the measurement approach itself and is analogous to estimating arterial Po_2_ from Hb saturation. Importantly, at lower $P\text{M}{\text{b}}_{{\text{O}}_{2}}$ values, the curve is steeper and provides greater sensitivity under the low-$P\text{M}{\text{b}}_{{\text{O}}_{2}}$ conditions characteristic of maximal exercise in the present study. Finally, the hyperbolic function used to characterize the ${\dot{\text{V}}\text{O}}_{2}$-$P\text{M}{\text{b}}_{{\text{O}}_{2}}$ relationship should be interpreted as a phenomenological description of mitochondrial O_2_ dependence rather than a definitive metabolic control model at the individual level. The linear P_50_ analysis was included as a complementary, nonmechanistic analysis to determine whether the primary interpretation depended on the assumed hyperbolic form. It should not be interpreted as a definitive metabolic control model. Similarly, comparisons with isolated mitochondrial measurements are intended primarily for physiological context, as substrate availability, energetic state, and metabolic regulation differ between the in vitro and in vivo studies.

### Conclusions

This study provides data-supported, proof-of-concept evidence that $P\text{M}{\text{b}}_{{\text{O}}_{2}}$, measured noninvasively using ^1^H-MRS, reflects in vivo ${\mathrm{Pmito}}_{{\text{O}}_{2}}$ during maximal exercise in humans. This interpretation is supported by low absolute $P\text{M}{\text{b}}_{{\text{O}}_{2}}$ values measured during maximal exercise, the expected relationship between muscle ${\dot{\text{V}}\text{O}}_{2}$ and $P\text{M}{\text{b}}_{{\text{O}}_{2}}$ across ${F\text{I}}_{{\text{O}}_{2}}$ conditions, and low apparent mitochondrial P_50_ values derived from both hyperbolic and linear analyses. Importantly, $P\text{M}{\text{b}}_{{\text{O}}_{2}}$ can be measured noninvasively using ^1^H-MRS. In the present study, separately acquired invasive measurements of muscle ${\dot{\text{V}}\text{O}}_{2\text{max}}$ were used only to establish the ${\dot{\text{V}}\text{O}}_{2}$-$P\text{M}{\text{b}}_{{\text{O}}_{2}}$ relationship. This approach may provide new opportunities to investigate the role of in vivo ${\mathrm{Pmito}}_{{\text{O}}_{2}}$ in muscle metabolism, adaptation, and physiological function in health and disease.

## Data Availability

Data will be made available upon reasonable request.
